# Next-Generation Antioxidants in Cardiovascular Disease: Mechanistic Insights and Emerging Therapeutic Strategies

**DOI:** 10.3390/antiox15020164

**Published:** 2026-01-25

**Authors:** Desh Deepak Singh, Dharmendra Kumar Yadav, Dongyun Shin

**Affiliations:** 1 Amity Institute of Biotechnology, Amity University Rajasthan, Jaipur 303002, India; ddsbms@gmail.com; 2 College of Pharmacy, Gachon University, Hambakmoeiro 191, Yeonsu-gu, Incheon 21924, Republic of Korea

**Keywords:** oxidative stress, mitochondria-targeted antioxidants, NADPH oxidase (NOX) inhibitors, Nrf2 activation, nanotechnology-based antioxidant delivery, cardiovascular disease prevention

## Abstract

Cardiovascular diseases (CVDs) remain the leading cause of mortality worldwide. CVDs are associated with multiple factors, including oxidative stress, mediated endothelial dysfunction, vascular inflammation, and atherothrombosis. Although traditional antioxidant supplementation (such as vitamins C, E, and β-carotene) has shown promising results in rigorous animal model studies, it has consistently failed to demonstrate clinical benefit in most human trials. Consequently, there is a substantial unmet need for novel paradigms involving mechanistically and biologically relevant pharmaceutical-grade antioxidant therapies (“next-generation antioxidants”). Rapid advancements in redox biology, nanotechnology, genetic modulation of redox processes, and metabolic regulation have enabled the development of new antioxidant therapeutics, including mitochondrial-targeted agents, NADPH oxidase (NOX) inhibitors, selenoprotein and Nrf2 activators, engineered nanoparticles, catalytic antioxidants, and RNA-based and gene-editing strategies. These interventions have the potential to modulate specific oxidative pathways that contribute to CVD pathogenesis. This review provides a comprehensive assessment of current oxidative stress–modulating modalities and their potential to inform personalized cardiovascular prevention and treatment strategies.

## 1. Introduction

Cardiovascular diseases (CVDs) continue to be the leading cause of mortality globally, accounting for an estimated 20.5 million deaths per year according to the World Health Organization [1]. CVDs include ischemic heart disease, stroke, hypertensive heart disease, peripheral arterial disease, heart failure, and other vascular-related conditions. Collectively, they account for more than one-third of deaths worldwide [2].

The increasing burden of CVDs is highlighted in numerous epidemiological assessments, including the Global Burden of Disease (GBD) report by Roth et al. [2], which demonstrates the persistent rise in cardiovascular risk across populations and health systems worldwide [2]. Rapid demographic transitions, such as ageing populations and increased life expectancy, combined with lifestyle factors including physical inactivity, smoking, and high consumption of calorie-dense foods, are contributing to a growing prevalence of cardiometabolic risk factors such as hypertension, type 2 diabetes, dyslipidaemia, and obesity [3].

Chronic low-grade inflammation and oxidative stress are global drivers of increased risk factors associated with endothelial dysfunction and atherogenesis. Oxidative stress has been identified as a key contributor to vascular injury; therefore, understanding how reactive oxygen species (ROS) generated by various enzymes contribute to this process is essential [4].

Research conducted by Madamanchi et al. and Harrison et al. has demonstrated that ROS play a central role in multiple biological processes associated with cardiovascular disease (CVD), including lipid peroxidation, depletion of nitric oxide, inflammatory responses, and plaque instability [5]. Evidence from these studies confirms that redox imbalance is a unifying mechanism across most CVD phenotypes. Consequently, greater emphasis should be placed on developing therapies that specifically target redox biology to address the underlying molecular mechanisms driving disease progression [6].

## 2. Oxidative Stress Pathways in Cardiovascular Disease

**Figure 1:**
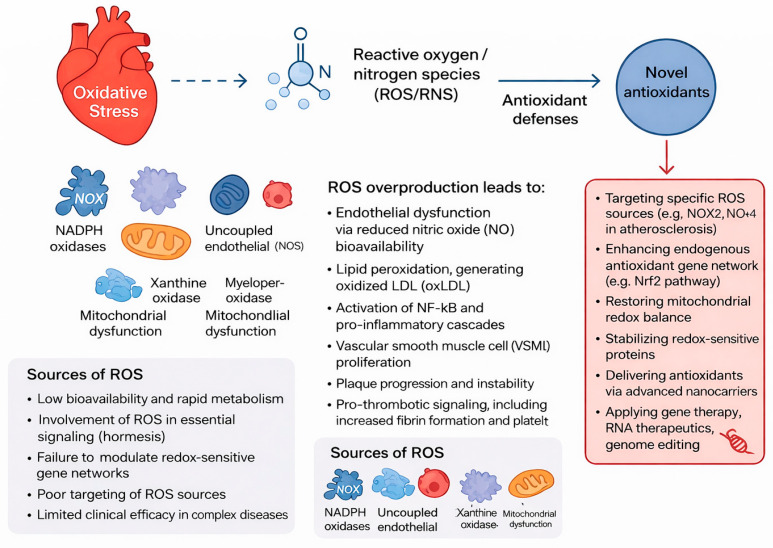
Oxidative Stress as a Central Driver of Cardiovascular Pathophysiology. Solid arrows indicate established mechanistic pathways, whereas the dashed line denotes an indirect or multistep association between oxidative stress and the generation of reactive oxygen and nitrogen species (ROS/RNS).

**Figure 2:**
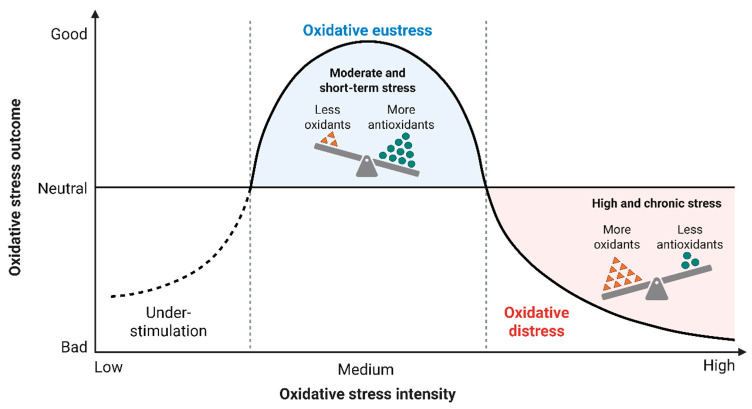
This figure illustrates the biphasic nature of oxidative stress, highlighting the transition from beneficial oxidative eustress to harmful oxidative distress. At low oxidative stress intensity, under-stimulation occurs, resulting in insufficient redox signaling and suboptimal cellular responses. Moderate and short-term increases in oxidants, balanced by adequate antioxidant defenses, produce oxidative eustress, which supports physiological redox signaling, adaptive responses, and cellular homeostasis. As oxidative stress intensity increases beyond the adaptive threshold, antioxidant capacity becomes overwhelmed. This shift leads to oxidative distress, characterized by high and chronic oxidant levels, diminished antioxidant defenses, and progressive cellular damage. Excessive oxidative stress promotes protein oxidation, lipid peroxidation, DNA injury, inflammation, and pathological signaling, ultimately contributing to tissue dysfunction and disease progression. The diagram emphasizes the importance of redox balance in maintaining cellular health and preventing oxidative pathology.

Oxidative stress is a key process involved in cardiovascular disease (CVD) development and clinical manifestation. Reactive oxygen species (ROS) are produced by several different enzymatic systems in the cell and help regulate vascular tone, inflammation, metabolic signaling, and cell survival (Figure 1) [7]. When too many ROS are produced for the body’s antioxidant capacity to handle, this imbalance (redox) can cause the cardiovascular system to undergo various structural and functional changes leading to disease [8]. Each of the major types of vascular cells (endothelium, ascular smooth muscle, cardiomyocyte, and immune cells) contains multiple molecular sources of ROS that contribute to disease progression [9]. The oxidant-antioxidant balance serves as a primary determinant of oxidative stress by disrupting cellular homeostasis and thereby causing tissue damage (Figure 2) [10]. As passive molecules created through oxidative stress, excess reactive oxygen and nitrogen species can exceed the body’s physiological antioxidant defense (superoxide dismutase, catalase, and glutathione), causing uncontrolled oxidation of lipids, proteins, and DNA [11]. Biochemical damage caused by these oxidized molecules reduces mitochondrial function, activates inflammatory pathways, and accelerates cellular aging and cell death (apoptosis). Further, the body’s antioxidant capacity can be reduced through inadequate intake of nutrients, exposure to chronic disease and inflammation, or genetic factors, increasing susceptibility to oxidative damage [12]. The biological response to sustained imbalance in oxidant-antioxidant levels has been implicated in the initiation and progression of CVD, neurodegenerative disease, metabolic disease, and inflammatory disease (Figure 1 and Figure 2) [13]. This narrative review was prepared in compliance with the SANRA guidelines, which ensure a high standard of quality and transparency. A comprehensive search of PubMed, Scopus, Embase, and Web of Science for articles published up to December 2025 was conducted. The following search terms were used: “next-generation antioxidants,” “cardiovascular disease,” “oxidative stress,” “reactive oxygen species,” “clinical trials,” and “statins.” Only studies published in English were considered.

Titles and abstracts of all retrieved articles were first screened, then a full-text review was carried out. Each study was evaluated for quality, methodology strength, and any potential biases, considering factors such as study design, sample size, and clarity of reported outcomes. Original mechanistic and clinical research articles were considered for inclusion along with review papers that provided valuable insights. Indeed, the evidence collected was synthesized to provide a structured overview of key topics: the mechanism of oxidative stress in CVD, strategies now emerging with next-generation antioxidants, considerations in human clinical trials, and potential benefits from combining these newer antioxidants with established cardiovascular therapies. This thus allowed us to give an all-inclusive and balanced perspective on current knowledge and future directions in the field.

This schematic illustrates the major cellular sources and pathological consequences of oxidative stress in cardiovascular disease. Excessive production of reactive oxygen and nitrogen species (ROS/RNS) arises from multiple enzymatic systems, including NADPH oxidases, uncoupled endothelial nitric oxide synthase (eNOS), xanthine oxidase, mitochondria, and myeloperoxidase. When ROS generation exceeds endogenous antioxidant defenses, redox imbalance leads to endothelial dysfunction, reduced nitric oxide (NO) bioavailability, lipid peroxidation with formation of oxidized LDL (oxLDL), activation of NF-κB–dependent inflammatory cascades, vascular smooth muscle cell (VSMC) proliferation, plaque progression, and pro-thrombotic signaling. The figure also summarizes the limitations of conventional antioxidant therapies, such as low bioavailability, rapid metabolism, and inability to modulate redox-sensitive gene networks. Novel antioxidant strategies aim to target specific ROS sources (e.g., NOX2), enhance endogenous antioxidant pathways (e.g., Nrf2 activation), restore mitochondrial redox balance, stabilize redox-sensitive proteins, and utilize advanced delivery platforms including nanocarriers, gene therapy, RNA-based therapeutics, and genome-editing approaches.

### 2.1. Sources of ROS in Cardiovascular Cells

#### 2.1.1. NADPH Oxidases (NOX)

RT is only one of many oxidoreductase enzymes that produce free radicals, primarily in the form of superoxide (O_2_^−^) [14]. Each isoform of NADPH oxidase (NOX) predominates in specific tissues, with several isoforms expressed at particularly high levels in vascular endothelial cells and smooth muscle [15]. All NOX isoforms contribute to oxidative stress–induced injury of the vascular endothelium and the development of hypertension, impairing normal vascular function and promoting atherosclerosis [16].NOX1 and NOX2 generate oxidized low-density lipoprotein (oxLDL), which recruits leukocytes and triggers the release of pro-inflammatory cytokines. Although NOX4 primarily generates hydrogen peroxide rather than superoxide, its chronic overexpression leads to cardiac and vascular fibrosis, diabetic cardiomyopathy, and maladaptive cardiac and vascular remodeling [17]. NOX5,present in humans but absent in rodents, plays a critical role in endothelial dysfunction, producing calcium-dependent oxidative bursts and promoting plaque destabilization, thereby contributing to the pathogenesis of acute coronary syndromes [18]. Taken together, the NOX family serves as a major initiator of oxidative signalling cascades that drive vascular inflammation and structural deterioration of the vasculature [19].

#### 2.1.2. Mitochondrial ROS

**Figure 3:**
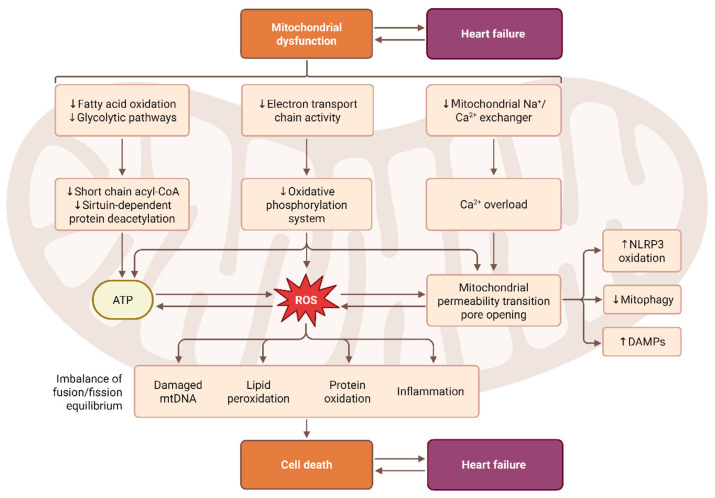
Mitochondrial Dysfunction–ROS Axis Linking Cellular Injury to Heart Failure. This schematic illustrates the interconnected pathways through which mitochondrial dysfunction contributes to the development and progression of heart failure. Impaired mitochondrial metabolism, including reduced fatty acid oxidation, glycolytic flux, short-chain acyl-CoA availability, and sirtuin-dependent protein deacetylation, leads to decreased ATP synthesis. Defects in the electron transport chain and oxidative phosphorylation promote excessive reactive oxygen species (ROS) generation. Mitochondrial Ca^2+^ overload, driven by impaired Na^+^/Ca^2+^ exchanger activity, further amplifies ROS production and triggers mitochondrial permeability transition pore (mPTP) opening. Elevated ROS levels result in oxidative damage to mitochondrial DNA, lipids, and proteins, as well as activation of inflammatory pathways. Reduced mitophagy, increased NLRP3 inflammasome activation, and enhanced release of mitochondrial damage-associated molecular patterns (DAMPs) exacerbate cellular stress. These maladaptive processes produce an imbalance in mitochondrial fusion–fission dynamics, ultimately leading to cell death. The resulting cardiomyocyte loss and energetic deficits create a vicious cycle that reinforces mitochondrial dysfunction and drives the progression of heart failure.

Mitochondria constitute another major source of ROS, particularly during oxidative phosphorylation [20]. Electron leakage from complexes I and III generates superoxide, which is rapidly converted into hydrogen peroxide [21]. Under physiological conditions, mitochondrial ROS serve important signaling functions; however, dysregulated mitochondrial metabolism, especially during sustained pressure overload, ischemia–reperfusion injury, or metabolic syndrome, dramatically increases ROS production [22].This leads to mitochondrial DNA damage, impaired ATP synthesis, opening of the mitochondrial permeability transition pore, and release of pro-apoptotic proteins. Excess mitochondrial ROS accelerates cardiomyocyte death, contributes to the progression of heart failure, and promotes vascular aging by impairing endothelial regeneration and nitric oxide signaling (Figure 3) [23].

#### 2.1.3. Uncoupled eNOS

Endothelial nitric oxide synthase (eNOS) is expected to generate nitric oxide (NO), a critical vasodilator and anti-inflammatory molecule [24]. However, insufficient availability of its cofactor tetrahydrobiopterin (BH4), a condition common in diabetes, hypercholesterolemia, and chronic inflammation, induces eNOS uncoupling [25]. In this state, eNOS produces superoxide rather than NO. This “reverse” enzyme activity not only reduces the bioavailability of NO but also increases oxidative load, contributing directly to endothelial dysfunction. Furthermore, superoxide combines with NO to form peroxynitrite, a highly reactive oxidant that oxidizes lipids, proteins, and DNA, exacerbating vascular injury [26].

#### 2.1.4. Xanthine Oxidase and Myeloperoxidas

Xanthine oxidase (XO) generates superoxide and hydrogen peroxide during purine metabolism. Its activity increases in hyperlipidemia, chronic heart failure, and ischemic conditions, contributing to oxidative endothelial injury and microvascular rarefaction [27]. Myeloperoxidase (MPO), derived from activated neutrophils and macrophages, promotes formation of hypochlorous acid, a potent oxidant that modifies LDL into highly atherogenic forms, impairs HDL function, and damages endothelial proteins [28]. Elevated MPO levels correlate with unstable plaques, systemic inflammation, and worse clinical outcomes [28].

### 2.2. Mechanistic Consequences of Redox Imbalance

Excessive ROS profoundly reshape cardiovascular physiology. One of the earliest consequences is endothelial dysfunction [29]. Superoxide reacts rapidly with NO, decreasing its bioavailability and forming peroxynitrite, which uncouples eNOS further and oxidizes critical lipid and protein targets [30]. Loss of NO signaling reduces vasodilation, increases vascular tone, and disrupts antithrombotic homeostasis. Chronic oxidative stress also promotes vascular inflammation [31]. ROS activate transcription factors such as NF-κB and AP-1, upregulating pro-inflammatory cytokines, adhesion molecules, and chemokines [32]. Additionally, ROS trigger NLRP3 inflammasome activation, promoting IL-1β maturation and amplifying sterile inflammation within atherosclerotic plaques and failing myocardium. Persistent inflammatory signaling accelerates endothelial injury and promotes a feed-forward loop of oxidative stress [33].

### 2.3. Role of Endothelial Dysfunction in Heart Failure

**Figure 4:**
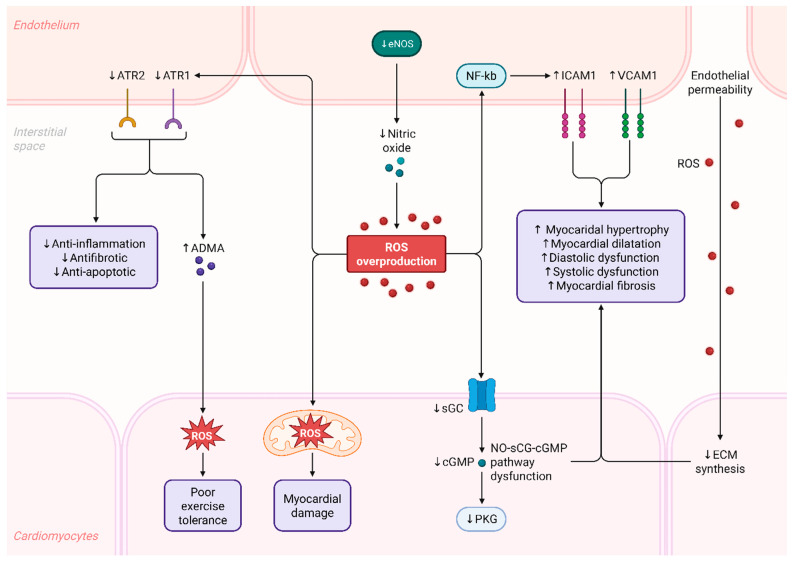
ROS-Mediated Endothelial and Myocardial Dysfunction in Cardiovascular Pathophysiology.

Vascular remodeling is another key outcome. ROS induces vascular smooth muscle cell migration, proliferation, and extracellular matrix deposition, processes central to hypertension-induced remodeling and neointimal formation [34]. Molecular mechanisms by which reactive oxygen species (ROS) mediate endothelial dysfunction and myocardial impairment. Reduced endothelial nitric oxide synthase (eNOS) activity lowers nitric oxide availability, promoting ROS overproduction and activating NF-κB–dependent inflammatory signaling. Upregulation of adhesion molecules (ICAM1, VCAM1) increases endothelial permeability, contributing to myocardial hypertrophy, dilation, systolic and diastolic dysfunction, and fibrosis. Alterations in angiotensin receptor signaling reduce anti-inflammatory and antifibrotic effects while elevated asymmetric dimethylarginine (ADMA) further suppresses nitric oxide generation. Excess ROS in cardiomyocytes leads to mitochondrial injury, ECM dysregulation, decreased sGC and cGMP signaling, impaired PKG activity, and downstream mechanical and metabolic dysfunction. These processes collectively result in myocardial damage, reduced exercise tolerance, and progressive cardiovascular deterioration (Figure 4). In atherosclerosis, oxidative stress fosters LDL oxidation, generating oxidized LDL (OxLDL), which drives macrophage uptake and foam cell formation. OxLDL also augments endothelial adhesion molecule expression and perpetuates plaque inflammation and growth [35]. Endothelial dysfunction plays a central role in the development and progression of heart failure. Healthy endothelial cells regulate vascular tone, maintain nitric oxide (NO) availability, and control inflammation and coagulation [36]. In heart failure, reduced NO production, increased oxidative stress, and heightened inflammatory signaling impair vasodilation and promote vascular stiffness [36]. This dysfunction leads to impaired coronary blood flow, increased afterload, and microvascular rarefaction, worsening myocardial oxygen delivery [37]. Additionally, endothelial activation enhances fibrosis, thrombosis, and neurohormonal imbalance, further deteriorating cardiac structure and function. Thus, endothelial dysfunction is both a trigger and an amplifier of heart failure pathophysiology, representing a key therapeutic target [38]. Finally, oxidative stress influences thrombosis. Increased ROS enhances platelet activation, reduces fibrinolysis by inactivating nitric oxide and prostacyclin pathways, and strengthens procoagulant signaling [39]. In advanced plaques, MPO and peroxynitrite destabilize fibrous caps, increasing the likelihood of plaque rupture and acute thrombotic events.

**Figure 5:**
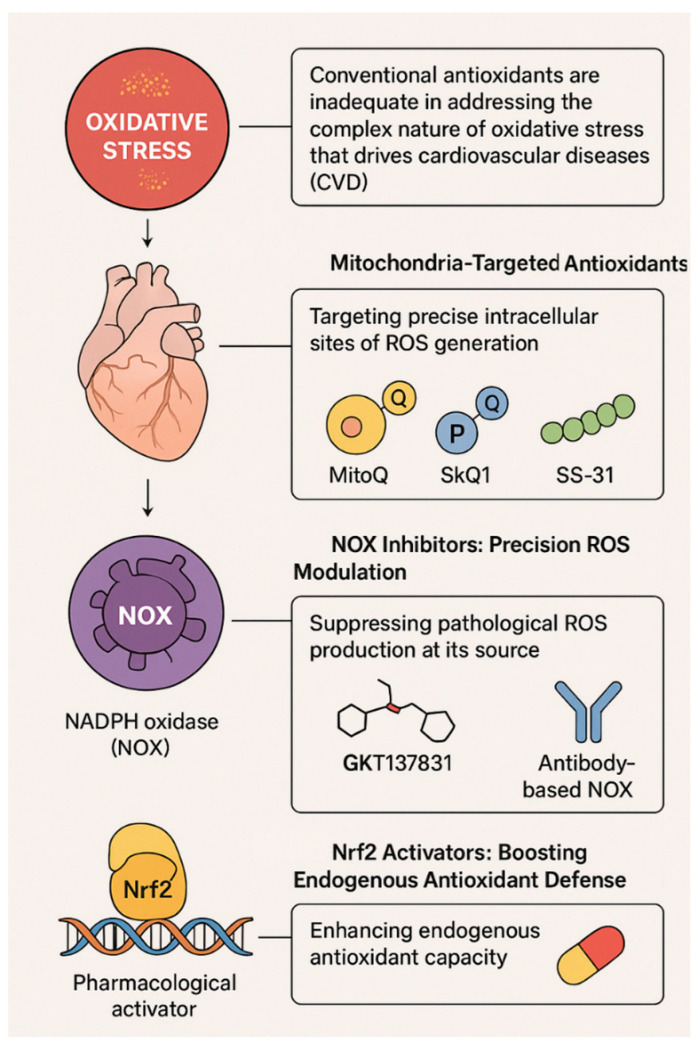
New Targeted Antioxidant Strategies for Cardiovascular Disease Treatment. This graphic reveals new, cutting-edge treatment strategies being developed to get around the shortcomings of traditional antioxidant treatments for cardiovascular disease (CVD). Targeted mitochondria antioxidants (i.e., MitoQ, SkQ1, and SS-31) work on ROS production right at their source in the mitochondria and improve mitochondrial function. NOX inhibitors provide precise regulation of pathological ROS formation by blocking NADPH oxidase activity and include small-molecule blockers like GKT137831 as well as anti-NOX modulators. Activators of the Nrf2 transcription factor increase endogenous antioxidant capacity by activating the Nrf2–ARE pathway (i.e., what happens when Nrf2 gets stimulated). Together, these strategies can provide superior ability to regulate the redox state of cells compared with traditional antioxidant treatments while specifically targeting the underlying oxidative stress pathways involved in CVD.

Long-term investigations into conventional antioxidants as a method for preventing CVD were driven by early epidemiological studies suggesting an inverse relationship between dietary antioxidant levels and cardiovascular risk [40]. However, numerous clinical trials evaluating conventional antioxidant supplementation, including vitamin E, vitamin C, β-carotene, and polyphenols, and their potential to provide cardiovascular protection have produced disappointing outcomes [41]. The overall lack of meaningful cardiovascular benefit observed in these trials indicates that conventional antioxidant supplementation does not adequately counteract the oxidative stress burden driven by localized, enzyme-mediated mechanisms involved in the development of CVD (Figure 5) [42].

### 2.4. Vitamin E

Alpha-tocopherol is one of the earliest antioxidants studied for the prediction of heart disease based on its properties to prevent lipid peroxidation by acting as a free radical scavenger for lipids in the body [43]. Initial studies investigating how vitamin E works at a cellular level indicated that vitamin E was able to prevent and reduce lipid peroxidation, thus allowing for preservation of the structure of cell membranes, causing an initial sense of optimism that perhaps vitamin E could help to decrease the risk of later developing atherosclerosis [44]. Clinical studies conducted later using large randomized clinical trials, such as the Heart Outcomes Prevention Evaluation (HOPE), Alpha-Tocopherol Beta-Carotene Cancer Prevention Study (ATBC) and the Supplementation en Vitamines et Minéraux Antioxydants (SU.VI.MAX), found that there was no significant decrease in the incidence of myocardial infarctions, strokes, or deaths from CV causes or death among the individuals included in those studies that received vitamin E supplementation [45]. In certain populations studied, the high doses of vitamin E taken led to an increased incidence of heart failure and/or hemorrhagic strokes among those receiving it. Based on this information, it appears that the ability of alpha tocopherol (i.e., vitamin e) to nonspecifically scavenge lipid peroxyl radicals does not effectively affect changes to the underlying pathophysiological mechanisms related to the oxidative stress that leads to cardiovascular disease (CVD) development [46].

### 2.5. Vitamin C

Vitamin C (ascorbic acid), a substantial water-soluble reductant, is capable of regenerating oxidized vitamin E and neutralizing various reactive species [47]. Vitamin C plays a key role in vascular homeostasis and vascular health when at normal levels; nevertheless, the clinical benefit of vitamin C supplementation has been disappointing in general [48]. The main reason for the limited clinical benefit derived from vitamin C is its poor transport into the mitochondria, the most abundant source of ROS production in endothelial and cardiac cells, resulting in the inability of vitamin C to restore oxidative damage to the mitochondria [48]. Because of this mechanism, it is not possible for vitamin C to restore or adequately protect against mitochondrial oxidative stress, which is known to result in endothelial dysfunction, ischemia–reperfusion injury, and progression of heart failure [49]. Additionally, the level of ascorbic acid in the blood is controlled through absorption in the intestines and excretion through the kidneys, thereby limiting how much vitamin C can reach sufficient intracellular concentrations through oral supplementation for therapeutic benefit [50]. Consequently, the results of clinical trials evaluating vitamin C (as a stand-alone or in conjunction with vitamin E) have demonstrated consistent failure in providing any significant level of cardiovascular protection, which has led to an increasing acceptance that nonspecific antioxidant supplementation does not address the localized, enzyme-modulated, redox processes involved in creating cardiovascular problems [51].

### 2.6. β-Carotene

Initially, β-carotene was considered a highly promising compound for cancer and cardiovascular disease (CVD) prevention and was widely tested in large clinical trials, but none demonstrated any meaningful benefit [52]. In fact, the ATBC and CARET trials revealed that β-carotene supplementation in smokers and individuals exposed to asbestos increased the incidence of lung cancer and overall mortality [53]. This unexpected harmful effect is thought to result from the tendency of β-carotene to become a pro-oxidant under conditions of high oxidative stress, generating toxic metabolites and harmful oxidative byproducts [54]. Moreover, β-carotene proved ineffective in reducing cardiovascular events, underscoring the importance of exercising caution when using antioxidants without considering the biochemical environment in which they function, their potential redox behavior, and the individual patient’s underlying risk profile [55].

### 2.7. Polyphenols

Polyphenols are a class of naturally occurring compounds that have received a great deal of attention because of their antioxidant capabilities, their ability to reduce inflammation, and their cardioprotective effects in preclinical studies [56]. Limited clinical trials have reported that dietary polyphenols can improve the endothelial function, lipid profiles, glucose metabolising process, and levels of inflammatory biomarker levels. Nevertheless, developing a protocol that provides consistent benefits from dietary polyphenols has proven to be difficult [57]. Most polyphenols have the disadvantage of very low oral bioavailability due to limited absorption from the gastrointestinal tract; extensive metabolism by the liver; and rapid clearance from the bloodstream following oral administration, leading to the presence of very low blood levels of polyphenols [58]. The active metabolites of many polyphenols are different from their parent compounds, making it difficult to interpret the mechanisms through which they function and accurately predict their effects [59]. All of these factors greatly reduce the concentration of polyphenols that can be delivered to target tissues, and therefore, prevent them from having therapeutic antioxidant activity, even though they have shown promising biological effects in research studies performed in controlled laboratory settings [60]. The results of numerous studies conducted over several years show a single, clear conclusion: nonspecific scavengers are not capable of correcting the complex, compartmentalized, and persistent dysfunction of the body’s ability to maintain a normal redox balance that contributes to the development of cardiovascular disease [61]. Antioxidants have been used extensively in research; however, they were found to be ineffective not since oxidative stress does not play an essential role in cardiovascular disease but because conventional antioxidant agent(s) cannot reach the area of the cell where the majority of abnormal production of reactive oxygen species takes place or regulate the major sources of production that are considered to be proximal to NOX, abnormal mitochondrial function and other dysfunctional organelles [62]. In addition, conventional antioxidant agents would inhibit other physiological signalling, hence the need for the development of next generation targeted antioxidant therapeutics that can specifically regulate oxidative stress and restore redox homeostasis while enabling the continued regulation of normal cellular signalling functions [63].

## 3. Next-Generation Antioxidant Therapies

Initially, β-carotene was considered a highly promising compound for cancer and cardiovascular disease (CVD) prevention and was widely tested in large clinical trials, but none demonstrated any meaningful benefit [64].Moreover, β-carotene proved ineffective in reducing cardiovascular events, underscoring the importance of exercising caution when using antioxidants without considering the biochemical environment in which they function, their potential redox behavior, and the individual patient’s underlying risk profile [65].

### 3.1. Mitochondria-Targeted Antioxidants

Pathological ROS (reactive oxygen species) production in endothelial dysfunction, heart failure, and ischemia–reperfusion injury arises primarily from mitochondria [66]. Traditional antioxidants do not effectively enter mitochondria or accumulate at concentrations sufficient to counteract mitochondrial ROS [67]. Consequently, researchers have developed mitochondria-targeted molecules that utilize lipophilic cations, mitochondria-penetrating peptides, or targeted nanocarriers to achieve selective mitochondrial delivery [68].

#### 3.1.1. MitoQ

MitoQ consists of a ubiquinone moiety covalently linked to the lipophilic cation triphenylphosphonium (TPP^+^). This molecular design enables MitoQ to selectively accumulate within mitochondria at concentrations 100 to 1000 times higher than in the cytosol, driven by the organelle’s highly negative membrane potential [69]. In preclinical studies, MitoQ supplementation consistently reduces mitochondrial ROS, enhances endothelial nitric oxide bioavailability, and decreases vascular inflammation. Human clinical trials have also demonstrated that MitoQ significantly improves endothelial function and markedly reduces arterial stiffness in older adults, supporting its translational potential for treating age-related vascular dysfunction [61].

#### 3.1.2. SkQ1 (Plastoquinonyl-Decyltriphenylphosphonium)

SkQ1 is a TPP^+^-conjugated antioxidant derived from plastoquinone that functions as an efficient scavenger of mitochondrial ROS [70]. It has demonstrated strong cardioprotective effects in animal models of ischemia–reperfusion injury. By stabilizing mitochondrial membranes, SkQ1 prevents mitochondrial permeability transition and thereby reduces cardiomyocyte apoptosis. Consequently, SkQ1 represents a promising therapeutic candidate for ischemia-induced cardiovascular diseases [71].

#### 3.1.3. SS-31 (Elamipretide)

Unlike MitoQ and SkQ1, SS-31 is a mitochondrial-penetrating tetrapeptide that binds specifically to cardiolipin. By preserving mitochondrial structure and inhibiting lipid peroxidation, SS-31 enhances ATP production, reduces ROS, and improves mitochondrial respiratory efficiency [72]. Early clinical studies have evaluated SS-31 in heart failure (HFpEF and HFrEF) and ischemia–reperfusion injury, positioning it as one of the most clinically advanced mitochondria-targeted therapies currently approved or under investigation [73].

### 3.2. NOX Inhibitors: Fine-Tuning ROS Production

NADPH oxidases (NOX enzymes) are uniquely positioned within the context of mammalian reactive oxygen species (ROS) generation, as no other mammal has such an exclusive goal and would thus be an obvious target for precision modulation of the redox state of cells [74]. In contrast to the byproduct nature of ROS produced as an unwanted byproduct of oxidative phosphorylation by the mitochondria, as well as by xanthine oxidase, the NOX isoforms (NOX1, NOX2, NOX4, and NOX5) function as tightly regulated enzymatic sources of superoxide and hydrogen peroxide produced by vascular and immune cells [75]. NOX-generated ROS are key contributors to cardiovascular disease due to their role in amplifying endothelial dysfunction, subsequent activation of NF-κB-mediated inflammation, mediated through impairment of nitric oxide signaling, and accelerating the development of an atherosclerotic plaque [76]. Thus, targeting the epicenter of pathological ROS production at the activator/source of production would effectively suppress the production of pathological ROS while leaving intact the production of beneficial physiological redox signals [76]. It is also important to note that each NOX isoform has a unique distribution within tissues, as well as distinct regulatory mechanisms, indicating that interventions will need to be designed to target an individual NOX isoform [77]. Recent advances in medicinal chemistry, structural modeling, and bioengineering of peptides have provided new opportunities for creating more selective inhibitors of NOX [78]. Precision inhibitors will ultimately have a tremendous impact on the prevention and/or treatment of oxidative vascular injury and represent an exciting new area of research in developing next-generation antioxidants [79].

#### 3.2.1. GKT137831 (Setanaxib)

Principally, the NOX4 isoform has a well-defined role in the progression of cardiovascular diseases [80]. Development and validation of an effective, selective NOX1/NOX4 inhibitor may also provide an effective treatment option for patients suffering from a variety of conditions associated with cardiovascular disease, such as heart failure, hypertensive cardiomyopathy (with preserved ejection fraction), and diabetic vascular disease [81]. In our body of research, setanaxib has demonstrated the ability to inhibit both vascular fibrosis and oxidative stress in vivo, and therefore it may serve as a potential disease-modifying agent to prevent or limit the effects of oxidative injury on the vasculature [82]. The findings from our research to date would support the conclusion that setanaxib will be of clinical significance for patients suffering from cardiovascular disease, and that further study with regard to its potential therapeutic use for these patients is warranted [83].

#### 3.2.2. Peptide and Antibody-Based NOX Inhibitors

Peptide and antibody-based NOX inhibitors are considered next-generation therapeutics, offering unprecedented isoform and interaction specificity [84]. A key example is the NOX2-ds-tat peptide, which is designed to block the p47^phox–NOX2 interaction required for NOX2 activation. This results in near-complete inhibition of NOX2-derived superoxide production while preserving other NOX isoforms and maintaining essential immune functions [85]. Likewise, emerging antibody-based inhibitors targeting NOX5, a calcium-dependent isoform present in human vasculature but absent in rodent models, provide a unique opportunity to modulate endothelial oxidative stress in a human-specific manner [86]. These biologics avoid many limitations associated with small-molecule inhibitors, such as off-target redox interactions or insufficient selectivity [87]. With high-affinity binding, structural precision, and the capacity to modulate extracellular or membrane-associated domains, peptide- and antibody-based inhibitors represent powerful candidates for advanced redox therapy [88]. Although these agents are still under development, their successful translation could revolutionize cardiovascular disease treatment through isoform-specific modulation of oxidative stress without disrupting physiological redox signaling [89].

### 3.3. Nrf2 Activators: Boosting Endogenous Antioxidant Defense

Nrf2 is a transcription factor that serves as the principal regulator of antioxidant and cytoprotective gene expression, including HO-1, NQO1, SODs, GPxs, and various Phase II detoxification enzymes [90]. Under basal conditions, Nrf2 is sequestered in the cytoplasm by KEAP1, which targets it for ubiquitin–proteasome–mediated degradation [91]. During oxidative or electrophilic stress, this interaction is disrupted, allowing Nrf2 to translocate into the nucleus, where it activates the transcription of genes involved in the cellular antioxidant response [92]. Pharmacologic Nrf2 activators enhance this endogenous defense system rather than merely scavenging reactive oxygen species; thus, they provide a sustained and physiologically aligned mechanism of protection [92]. In cardiovascular disease, Nrf2 activation reduces endothelial inflammation, increases nitric oxide bioavailability, and improves mitochondrial function, partly by decreasing oxidative damage and reducing the risk of atherosclerotic plaque rupture [93]. However, achieving therapeutic benefit requires careful modulation, as excessive Nrf2 activation or off-target metabolic effects may pose risks. Despite these challenges, Nrf2 activators remain a highly promising class of next-generation therapeutics.

Early clinical studies indicate that SS-31 may improve functional capacity in patients with heart failure with preserved ejection fraction (HFpEF) and reduced ejection fraction (HFrEF), and it appears to mitigate injury associated with ischemia–reperfusion [94]. Owing to its unique mechanism and the promising human data reported to date, SS-31 is considered one of the most advanced mitochondria-targeted therapies under development for cardiovascular disease [95].Next-Generation Antioxidant Therapies: Agents, Types, Mechanisms, Key Evidence, and Considerations are summarized in Table 1.

## 4. Clinical Evidence and Translational Progress of Next-Generation Antioxidants

Next-generation antioxidant medicines are being developed to bridge the gap between laboratory discoveries and clinical application in cardiovascular medicine [102]. Although oxidative stress has long been recognized as a key contributor to endothelial dysfunction, early clinical studies using conventional antioxidants produced limited benefits [103]. This has driven significant scientific interest in identifying and developing more precisely targeted therapies [104]. In recent years, substantial progress has been made with mitochondria-targeted molecules, NOX inhibitors, Nrf2 activators, and nanotechnology-based delivery systems, several of which have now advanced into human clinical trials [105]. While variability in clinical outcomes still exists across these therapeutic classes, the growing body of evidence strongly suggests that redox signaling can be modulated with high specificity, offering measurable and clinically meaningful benefits for cardiovascular disease [106].

### 4.1. MitoQ Clinical Trials

MitoQ is one of the leading mitochondrial antioxidants with the highest level of clinical advancement [107]. Early-phase trials conducted in older adults demonstrated significant improvements in endothelial function, accompanied by reductions in mitochondrial ROS and increased nitric oxide availability. Additionally, MitoQ supplementation led to notable reductions in arterial stiffness, a well-established risk factor for cardiovascular events, supporting its role in protecting against vascular aging and preventing age-related diseases [108]. Studies in individuals with nonalcoholic fatty liver disease (NAFLD) also showed reduced hepatic inflammation and oxidative stress, suggesting broader systemic benefits that may further lower the risk of cardiovascular disease [109]. Although large multicenter outcome trials are still lacking, MitoQ’s favorable safety profile and consistent improvements in vascular function highlight its potential as a promising long-term therapeutic agent for cardiometabolic disorders [110].

### 4.2. Elamipretide Trials

Elamipretide (SS-31) represents another important advancement in mitochondria-targeted therapeutics [111]. Clinical trials in patients with heart disease or heart failure have demonstrated increases in left ventricular stroke volume and improvements in mitochondrial energy production, providing proof of concept that peptide-based therapies can restore cardiomyocyte bioenergetics [112]. Early-phase studies further confirm that mitochondrial dysfunction is now considered a treatable pathological mechanism rather than merely a downstream indicator [113]. Ongoing larger clinical trials in mitochondrial myopathy and heart disease are evaluating additional benefits and clinical endpoints, potentially including measures of cardiac function in older adults with heart failure [114]. The expanding body of evidence supports the potential of Elamipretide as an effective therapeutic option for conditions associated with impaired mitochondrial energy production, including both heart failure with preserved ejection fraction and heart failure with reduced ejection fraction [115].

### 4.3. Setanaxib Trials

As a selective NOX1/NOX4 inhibitor, Setanaxib (GKT137831) has emerged as a promising candidate for redefining the treatment of fibrotic and inflammatory diseases [116]. Clinical trials conducted to date have shown that patients with chronic kidney disease and those with primary biliary cholangitis experience meaningful reductions in tissue fibrosis and oxidative stress following Setanaxib treatment [117]. Although these studies have not specifically targeted cardiovascular disease, the strong link between NOX-derived ROS and endothelial injury, vascular inflammation, and fibrosis underscores the important translational implications of these findings [118]. Furthermore, several ongoing and planned studies are evaluating Setanaxib’s effects on arterial stiffness, vascular inflammation, and the progression of atherosclerosis, which will provide additional insight into its cardiovascular safety and therapeutic potential [119]. Given its favorable safety profile to date and its highly specific inhibition of enzymatic NOX-generated ROS, Setanaxib is positioned to become the first validated precision NOX inhibitor for human disease [120].

### 4.4. Findings on the Function and Potential Applications of Nrf2 Activators

A variety of compounds have been identified as potential Nrf2 activators, including bardoxolone methyl, sulforaphane, and dimethyl fumarate. These agents may reduce oxidative stress and chronic inflammation by enhancing endogenous antioxidant defenses [121]. Clinical experience with these compounds has yielded mixed results: bardoxolone, for instance, showed early protective effects on renal tissue but was also associated with adverse events such as fluid retention and an increased risk of heart failure in certain patients [122]. This illustrates the need to balance the broad cytoprotective actions of Nrf2 activation with the potential risks associated with indiscriminate, systemic pathway stimulation [123]. Dimethyl fumarate has demonstrated vascular anti-inflammatory effects; however, additional studies specifically examining cardiovascular outcomes are still required [124]. Collectively, these findings highlight the need for next-generation Nrf2 activators capable of achieving tissue-specific modulation while minimizing excessive activation of downstream pathways throughout the body [125].

### 4.5. Nanomedicine Evaluation Trials

Nanotechnology-based antioxidant delivery approaches are still in their early stages, with most current human studies focused primarily on assessing tolerability, biodistribution, and pharmacokinetic profiles [126]. Early investigations using liposomal and polymeric carriers have demonstrated good tolerability; however, concerns regarding chronic accumulation, immune system activation, and limited specificity continue to restrict their clinical application [127]. Despite these challenges, substantial evidence shows that ROS-responsive and mitochondria-targeted nanoparticle systems can produce significant therapeutic effects in preclinical models of cardiovascular disease by improving drug stability, bioavailability, and target engagement [128]. Broad adoption into large-scale human trials will depend on advancements in nanoparticle safety engineering, standardization of manufacturing processes, and the establishment of clearer regulatory pathways for these emerging therapeutic platforms [129].Clinical evidence and translational progress of next-generation antioxidants in cardiovascular therapy are summarized in Table 2.

## 5. Integration of Antioxidants into the Cardiovascular Therapeutic Framework

The implementation of next-generation antioxidants in CVD treatment requires more than simply providing generic supplementation; it demands a mechanistic, evidence-based therapeutic approach [135]. CVDs are now recognized as conditions driven by chronic oxidative stress, mitochondrial dysfunction, and impaired redox signaling. Early research with vitamin C and vitamin E yielded poor outcomes due to non-specific treatment strategies, low bioavailability, and limited understanding of the complexities of redox biology [135]. In contrast, next-generation antioxidants, designed to target specific pathways such as NOX enzymes and mitochondrial dysfunction, offer far greater precision than traditional antioxidants [136]. The successful integration of these novel agents will rely on the rational combination of targeted antioxidants with existing pharmacological therapies, as well as the continued advancement of precision medicine using carefully selected biomarkers [137].

### 5.1. Combining Antioxidants with Standard Cardiovascular Therapies

Standard cardiovascular disease (CVD) medications, such as statins, ACE inhibitors, angiotensin receptor blockers (ARBs), beta-blockers, and sodium–glucose cotransporter 2 (SGLT2) inhibitors, have numerous pleiotropic benefits, including effects not directly related to blood flow dynamics [138]. Notably, many of these medications also reduce oxidative stress and therefore exert multiple therapeutic effects on both the cardiovascular system and the rest of the body [139]. A common strategy in CVD management is the combined use of standard medications with next-generation antioxidants. For example, statins not only lower LDL cholesterol but also reduce vascular superoxide production and enhance the activity of endothelial nitric oxide synthase (eNOS) [140]. When paired with mitochondria-targeted antioxidants (MTAs) such as MitoQ or SS-31, statins can further improve endothelial function, help restore mitochondrial membrane potential (MMP), and limit the formation of oxidized LDL [141]. Increased levels of mitochondrial proteins involved in lipid metabolism further enhance lipid homeostasis, complementing the lipid-modulating effects of statins [142]. ACE inhibitors also exhibit pleiotropic effects by reducing plasma angiotensin II levels, thereby inhibiting NOX activation and decreasing ROS production [143]. Direct NOX inhibitors, such as setanaxib (GKT137831), further suppress ROS generation by blocking the enzyme’s catalytic activity. Therefore, combining these therapeutic classes has the potential to significantly improve microvascular health in individuals with hypertension and/or heart failure [144].

SGLT2 inhibitors play an important role in the treatment of heart failure and diabetic cardiomyopathy because they reduce systemic oxidative stress through enhanced ketone metabolism, reduced inflammation, and improved mitochondrial redox efficiency [145]. When combined with antioxidants that stabilize mitochondrial membranes, patients with metabolic cardiovascular disease (CVD), heart failure with preserved ejection fraction (HFpEF), and/or obesity-related endothelial dysfunction may experience even greater therapeutic benefit from both classes of drugs [146].

It is important to consider pharmacokinetics and redox balance when designing combination therapies. Excessive suppression of reactive oxygen species (ROS) can disrupt normal physiological signaling pathways; therefore, these therapies must be administered in alignment with their specific mechanisms of action and appropriately titrated [147]. Early studies have shown that combination treatment strategies result in better outcomes than either agent alone; however, larger long-term clinical trials are still needed to determine the most effective combinations and to establish long-term safety [148].

The integration of biomarkers obtained through advanced imaging, multi-omics analyses, and genetic profiling allows clinicians to establish a unique redox phenotype for each individual and use this information to tailor antioxidant therapy [149]. This precisely guided approach enables antioxidants to be applied as targeted therapeutic agents rather than general nutritional supplements, thereby enhancing their effectiveness when used alongside current cardiovascular drugs while minimizing off-target effects [150]. Ultimately, biomarker-driven interventions will support more personalized management of cardiovascular disease, improving patient outcomes by addressing the specific oxidative and inflammatory pathways driving disease progression [151].

### 5.2. Integration of Antioxidants Using Precision Medicine Tools

Precision medicine has emerged as an important approach within cardiovascular care, enabling the use of antioxidant therapies guided by biomarkers of oxidative stress, mitochondrial dysfunction, and inflammation [152]. Unlike previous “standardized” intervention strategies, precision medicine allows clinicians to identify patients who are most likely to benefit from targeted antioxidant therapy (Table 2) [153].

The biomarker 8-iso-PGFα reflects lipid peroxidation and systemic oxidative stress, helping clinicians identify patients who may benefit from mitochondria-targeted antioxidants or NADPH oxidase (NOX) inhibitors [154]. Elevated oxidized low-density lipoprotein (oxLDL) levels indicate ongoing oxidative modification of lipoproteins and suggest that the patient may respond favorably to therapies that reduce mitochondrial reactive oxygen species (ROS) production or enhance high-density lipoprotein (HDL)-mediated reverse cholesterol transport [155].

Myeloperoxidase (MPO) levels indicate oxidative injury driven by inflammation and serve as markers of plaque vulnerability [156]. Furthermore, profiling different NOX isoforms allows clinicians to distinguish between NOX1-, NOX2-, and NOX4-mediated ROS production, enabling the selection of appropriate selective NOX inhibitors, such as setanaxib, for more precise and effective treatment [157]. Table 3 summarizes the integration of antioxidants into cardiovascular therapeutics, including strategies, underlying mechanisms, and clinical considerations.

## 6. Clinical Trials on Next-Generation Antioxidants in Cardiovascular Disease

Oxidative stress represents one of the major pathological mechanisms participating in the development and progression of CVD, but large-scale clinical studies on its modulation with classical antioxidant supplements have failed and been inconclusive [165]. Such failure may be explained by the absence of nonspecific oxidative stress scavenging, effective dosing modalities, and patient stratification, and, importantly, by the unavailability of any meaningful and quantitative estimate of oxidative stress before and following treatment administration [166]. On the other hand, the next generation of antioxidant agents target selectively particular redox mechanisms, cellular sites, or enzymes involved in ROS generation, and this specificity suggests new perspectives of potential clinical applicability [167,168]. Anyway, if one wishes to obtain any information on their efficacy, in future clinical studies, it will be mandatory to include basic key features of oxidative stress level evaluation as recommended by expert recommendations [169,170]. Validated biomarkers include circulating lipid peroxidation products, oxidized low-density lipoprotein (oxLDL), redox-sensitive inflammatory markers, and total antioxidant capacity indices that represent feasible and clinically relevant tools for the estimation of oxidative burden [171,172]. Longitudinal measurement of these biomarkers at baseline and at predefined follow-up intervals is important to capture the dynamic nature of changes in redox status and to establish pharmacodynamic responses to therapy [167]. In addition to biochemistry, trial designs should also encompass targeted redox endpoints and traditional cardiovascular outcomes to improve their sensitivity in detecting therapeutic response. Endothelial function, arterial stiffness, inflammatory mediators, lipid peroxidation, and clinical events MACE can be measured in tandem with oxidative stress biomarkers [173,174]. Integrated approaches will help clinicians and scientists decipher whether their oxidative stress modulation has paralleled their vascular and clinical efficacy benefits [173]. Of high importance in these considerations should be the theoretically modified or combined effects of conventional cardiovascular therapeutic agents, with particular attention devoted to the role of statins in combination with new generations of antioxidant agents [174]. In addition to lipid-lowering effects, statins also modulate endothelial cell function, inflammation, and ROS production [175,176]. Targeted antioxidant therapies could potentially further amplify such effects by selectively inhibiting the pathological production of ROS, rather than redox signaling, thus potentially augmenting the levels of available nitric oxide and vascular protection, respectively, by redox-sensitive signaling, as suggested in reference [177]. In this respect, therefore, combination therapy protocols combining existing pharmacologic therapies with novel antioxidant agents might constitute a potentially more clinically feasible and efficacious strategy, as a post-trial concern for combination therapy studies using existing pharmacologic agents in CVD [177]. In this respect, therefore, and for the future, clinical trials combining redox biomarkers and redox signaling in CVD will be the critical success factors for the future clinical translation of next-generation antioxidant agents in CVD, as proposed in reference [4,10]. Key Recommendations for Advanced Clinical Trial Design to Evaluate Next-Generation Antioxidants in Cardiovascular Disease are shown in Table 4.

## 7. Challenges and Future Directions

Reactive oxygen species (ROS) exert both beneficial and harmful effects on the cardiovascular system; therefore, developing therapies that modulate ROS levels remains a central challenge in cardiovascular research [178]. This duality necessitates a precise understanding of the specific ROS sources, including distinct enzyme isoforms, their cellular and tissue localization, and the mechanisms through which they drive injury and inflammation [179]. Inadequate targeting of these pathways risks disrupting essential physiological processes such as vasodilation, immune defense, and adaptive mitochondrial signaling [180].

Another major challenge is safety and bioavailability. Many promising antioxidants, such as polyphenols, mitochondria-targeted molecules, and peptide-based therapies, are limited by poor solubility, rapid metabolism, and insufficient tissue penetration [181]. Nanocarriers offer a potential solution by improving drug delivery, enhancing stability, and enabling controlled release at sites of oxidative stress [182]. However, they also introduce concerns related to long-term safety, pharmacokinetics, and immune activation, which must be rigorously evaluated in early-phase clinical studies [182,183].Therapeutic development is further complicated by species differences between humans and common laboratory animals, especially concerning NOX isoforms. For example, NOX5 is present in humans but absent in rodent models, underscoring the limitations of conventional preclinical systems [183]. This highlights the need for humanized disease models or ex vivo platforms supported by biomarker analysis to more accurately assess therapeutic efficacy and safety [184]. Precision medicine, guided by biomarkers such as oxidized LDL, 8-iso-PGF2α, and NOX isoform profiles, provides a framework for identifying patients most likely to benefit from antioxidant therapies and for designing individualized treatment strategies [185].

Gene therapy and RNA-based approaches also hold promise for correcting redox dysregulation at its molecular origins [186]. CRISPR/Cas9-mediated repair of eNOS uncoupling, mitochondrial DNA mutations, or NOX overexpression, as well as AAV-mediated enhancement of key antioxidant enzymes such as catalase, SOD2, or GPx1, represent emerging strategies [187]. When combined with pharmacological or nanoparticle-delivered antioxidants, these interventions could shift management from symptom control to true disease modification, offering durable and tissue-specific regulation of harmful ROS [188]. Nevertheless, careful assessment of off-target genetic effects, vector immunogenicity, and long-term safety remains essential.

Finally, the integration of digital health technologies, such as wearable sensors, non-invasive vascular imaging, and real-time oxidative stress monitoring, offers a transformative opportunity for adaptive antioxidant therapy [189]. Dynamic feedback on endothelial function, vascular stiffness, and systemic oxidative burden will enable individualized dosing protocols and quicker identification of therapeutic failure, while minimizing adverse events [190]. Realizing the full potential of next-generation antioxidants in cardiovascular medicine will require coordinated advancements in mechanistic precision, delivery technologies, translational modeling, genetic approaches, and digital health integration [190].

## 8. Limitations of the Study

Despite the growing interest in next-generation antioxidants for cardiovascular disease, several limitations should be acknowledged [165]. Much of the mechanistic evidence is derived from preclinical and experimental models, which may not fully replicate the complexity of the regulation of oxidative stress and redox signaling in human cardiovascular disease [166,178]. Translational gaps exist between molecular findings and clinically meaningful outcomes. Second, clinical trials to date have demonstrated significant heterogeneity in antioxidant type/formulation, dosing, treatment duration, and study populations [167,179]. This makes direct study comparisons challenging and limits the confidence with which one can conclude about efficacy [168,180]. Dosing strategies for many next-generation antioxidants have not been settled, creating risks of underdosing, or loss of redox poise at higher dosing levels [169,181].

The third major limitation in existing studies is the inconsistent assessment of oxidative stress; most trials have used indirect or single-time-point biomarkers rather than validated, repeatable, and longitudinal measures of reactive oxygen species or redox status [191]. This impairs the confirmation of target engagement and thus the correlation between biochemical effects with clinical endpoints [191].

Fourth, variability in clinical outcomes persists because oxidative stress is just one component of the multifactorial pathophysiology in cardiovascular disease [192]. The relationship between oxidative stress, inflammation, metabolic dysfunction, and endothelial signaling is bidirectional and far from simple; antioxidant therapy, in turn, may alone be unable to alter hard cardiovascular outcomes in certain populations [192].

Finally, there is a possibility of publication bias and selection bias, as this is a narrative review. Moreover, only English language articles were included in this review. Although structured guidelines were followed to make the review as rigorous as possible, the findings must be interpreted in the context of these inherent limitations.

## 9. Conclusions

Next-generation antioxidant therapies represent a breakthrough in the treatment and prevention of cardiovascular disease (CVD). While traditional antioxidants rely on nonspecific scavenging of reactive oxygen species (ROS) and therefore offer limited clinical benefit, modern strategies are designed to target the specific origins and mechanisms of oxidative stress. These advanced therapies focus on mitochondrial dysfunction, isoform-specific ROS generation by NOX enzymes, and impaired endogenous antioxidant defenses. By addressing pathological redox signaling while preserving essential physiological ROS functions, these approaches offer more effective support for patients with cardiovascular disease.

Emerging evidence supports the use of mitochondria-targeted small molecules such as MitoQ and SS-31, which have demonstrated improvements in endothelial function, arterial stiffness, and mitochondrial bioenergetics. Selective NOX inhibitors like setanaxib enable isoform-specific suppression of enzymatic ROS production, while Nrf2 activators and catalytic antioxidants strengthen intrinsic cytoprotective mechanisms. Gene- and nanotechnology-based systems, including CRISPR/Cas9 editing, AAV-mediated delivery of antioxidant enzymes, and nanocarrier-enabled targeted delivery, offer the potential for durable, tissue-specific redox modulation with improved bioavailability and safety.

Integrating digital health technologies with biomarker-guided precision medicine further enhances personalized therapy optimization. Although large-scale outcome trials are ongoing, current evidence increasingly supports shifting from broad antioxidant supplementation to mechanism-driven, targeted redox modulation. Collectively, these innovations position next-generation antioxidants at the forefront of precision cardiology.

## Figures and Tables

**Table 1:** Next-Generation Antioxidant Therapies: Agents, Types, Mechanisms, Key Evidence, and Considerations.

S.N.	Agent	Type	Mechanism	Key Evidence	Considerations	References
1	MitoQ	Mitochondria-targeted antioxidant	TPP^+^-driven mitochondrial uptake; reduces mito-ROS	Human trials: improved endothelial function, reduced arterial stiffness	Long-term CV benefit yet unconfirmed	[96]
2	SkQ1	Mitochondria-targeted antioxidant	Plastoquinone-TPP^+^ conjugate; prevents cardiomyocyte apoptosis	Strong cardioprotection in I/R injury models	Clinical translation ongoing	[97]
3	SS-31 (Elamipretide)	Mitochondrial peptide	Binds/stabilizes cardiolipin; improves ETC efficiency	HFpEF & HFrEF early trials show benefit	Delivery optimization needed	[98]
4	GKT137831 (Setanaxib)	NOX1/NOX4 inhibitor	Suppresses fibro-inflammatory ROS pathways	Reduces oxidative stress and vascular fibrosis	Few CV-focused trials completed	[99]
5	NOX2-ds-tat peptide	Peptide NOX2 inhibitor	Blocks p47phox–NOX2 assembly	Specific suppression of NOX2 superoxide	Early-stage; delivery challenges	[100]
6	NOX5 antibodies	Antibody inhibitor	Neutralizes NOX5 activity (human-specific)	Strong rationale for endothelial ROS control	Preclinical; stability/delivery issues	[101]
7	Nrf2 activators	Endogenous antioxidant boosters	KEAP1 inhibition → activation of cytoprotective gene network	Improved endothelial function & mitochondrial resilience	Overactivation risk	[101]

**Table 2:** Clinical Evidence and Translational Progress of Next-Generation Antioxidants in Cardiovascular Therapy.

S.N.	Therapeutic Class/Agent	Clinical Trial Evidence	Key Cardiovascular Effects	Stage of Clinical Translation	References
1	Mitochondria-Targeted Antioxidants	MitoQ: improved endothelial function and reduced arterial stiffness in older adults; reduced liver inflammation in NAFLD	Enhanced endothelial NO bioavailability; reduced mitochondrial ROS; potential reduction in vascular aging and cardiometabolic risk	Early- to mid-phase human trials; larger CVD outcome studies pending	[130]
2	Elamipretide (SS-31)	Improved left ventricular stroke volume; ongoing trials in mitochondrial myopathy and heart failure	Restores mitochondrial energetics; reduces ROS; improves cardiac contractility	Phase II–III trials ongoing	[131]
3	NOX Inhibitors (Setanaxib/GKT137831)	Reduced fibrosis biomarkers in renal and fibrotic diseases; CVD-focused trials planned	Isoform-specific ROS suppression; improved endothelial function; potential anti-atherosclerotic effect	Early translational stage; cardiovascular trials in planning	[132]
4	Nrf2 Activators	Bardoxolone methyl: mixed results; dimethyl fumarate shows vascular anti-inflammatory effects	Enhances endogenous antioxidant defenses; reduces oxidative stress	Early human trials; limited by off-target effects	[133]
5	Nanomedicine-Based Antioxidants	Liposomal or polymeric carriers tested for safety and biodistribution	Improved antioxidant stability, bioavailability, and targeting	Early clinical studies; preclinical efficacy robust	[134]

**Table 3:** Integration of Antioxidants into Cardiovascular Therapeutics: Strategies, Mechanisms, and Clinical Considerations.

S.N.	Strategy/Approach	Mechanism/Rationale	Potential Benefits	Challenges/Considerations	Key References
1	Combination with Standard Therapies	Statins, ACE inhibitors/ARBs, beta-blockers, SGLT2 inhibitors possess intrinsic antioxidant or redox-modulating effects; combining with targeted antioxidants may enhance efficacy	Synergistic reduction in oxidative stress; improved endothelial function; enhanced mitochondrial protection; reduced arterial stiffness	Requires careful dosing to avoid excessive ROS suppression; potential pharmacokinetic interactions	[158]
2	Mitochondria-Targeted Antioxidants with Statins or SGLT2i	MitoQ, SS-31, SkQ1 restore mitochondrial membrane potential and NO bioavailability	Improved vascular and cardiac function; reduced oxidative damage in aging and metabolic CVD	Long-term safety and large-scale clinical outcome data needed	[159]
3	NOX Inhibitors with RAAS Modulation	Setanaxib or peptide-based NOX inhibitors + ACE inhibitors/ARBs	Dual suppression of enzymatic ROS; reduced vascular fibrosis and endothelial dysfunction	Species differences in NOX isoforms; careful patient selection via biomarkers	[160]
4	Biomarker-Guided Precision Therapy	Use of 8-iso-PGF_2_α, OxLDL, MPO, NOX isoform profiling to tailor antioxidant therapy	Personalized therapy targeting specific oxidative pathways; improved efficacy; minimized off-target effects	Requires validated biomarkers; standardization across labs; cost considerations	[161]
5	Integration with Multi-Omics and Genetic Profiling	Combining metabolomics, proteomics, genomics with redox biomarkers	Identification of patient-specific redox phenotypes; targeted interventions for high-risk populations	Complex data interpretation; requires specialized infrastructure	[162]
6	Digital Health and Continuous Monitoring	Wearable devices and sensors to track endothelial function, arterial stiffness, oxidative stress	Dynamic adjustment of therapy; early detection of therapy failure; improved adherence	Technology validation; data privacy; integration into clinical workflow	[163]
7	Overall Integration	Multi-modal approach combining pharmacologic antioxidants, gene therapy, nanocarriers, standard drugs, and biomarker guidance	Optimized, personalized redox modulation; improved CVD outcomes; reduced adverse events	Requires robust clinical trials; regulatory approval; interdisciplinary coordination	[164]

**Table 4:** Key Recommendations for Advanced Clinical Trial Design to Evaluate Next-Generation Antioxidants in Cardiovascular Disease.

S.N.	Design Element	Recommended Strategy	Scientific and Clinical Rationale	References
1	Study population	Stratification based on baseline oxidative stress status	Improves patient selection and enhances the likelihood of detecting treatment effects	[165,166,167,168,169,170]
2	Antioxidant class	Use of targeted or pathway-specific antioxidants (e.g., mitochondria-targeted antioxidants, NOX inhibitors, Nrf2 modulators)	Avoids nonspecific ROS suppression and preserves physiological redox signaling	[168,169,174]
3	Dose selection	Biomarker-guided or adaptive dosing strategies	Accounts for dose-dependent effects and interindividual variability in redox balance	[166,174]
4	Oxidative stress assessment	Inclusion of validated, easily repeatable biomarkers (e.g., oxidized LDL, malondialdehyde, 8-iso-prostaglandin F2α, total antioxidant capacity)	Enables objective evaluation of drug-induced modulation of oxidative stress	[165,171,172,173,174]
5	Timing of biomarker measurement	Baseline and longitudinal assessment during and after intervention	Captures dynamic changes in redox status in response to therapy	[169,174]
6	Combination therapy	Evaluation of next-generation antioxidants in combination with standard cardiovascular drugs	Reflects real-world clinical practice and may enhance therapeutic efficacy	[174,175]
7	Statin–antioxidant interaction	Assessment of pleiotropic statin effects on oxidative stress and endothelial function	Statins reduce ROS generation and may synergize with targeted antioxidants	[175,176,177]
8	Clinical endpoints	Integration of redox biomarkers with conventional cardiovascular outcomes	Strengthens mechanistic interpretation and clinical relevance	[167,173,174]
9	Trial duration	Adequate follow-up to observe biochemical and vascular effects	Short-term trials may underestimate antioxidant benefits	[166,172,174]
10	Precision medicine approach	Adaptive or personalized trial designs based on oxidative stress response	Supports individualized therapy and improves translational success	[168,174]

## Data Availability

No new data were created or analyzed in this study.
